# Regulating Memory Use: Individual Differences in Working Memory Capacity in Natural Tasks

**DOI:** 10.3390/bs16081444

**Published:** 2026-08-21

**Authors:** Xutao Zheng, Mowei Shen, Hui Chen, Jifan Zhou

**Affiliations:** 1 Department of Psychology and Behavioral Sciences, Zhejiang University, Hangzhou 310058, China; zhengxutaowk@foxmail.com; 2 Zhejiang Key Laboratory of Neurocognitive Development and Mental Health, Zhejiang University, Hangzhou 310058, China

**Keywords:** working memory capacity, voluntary memory use, attentional control, individual differences, copying task

## Abstract

Working memory (WM) capacity predicts a wide range of cognitive abilities, yet little is known about how individuals with different WM capacities regulate memory use during natural tasks in which memory use is voluntarily determined. To address this question, we conducted two experiments (N = 120) in which participants completed a copying task and a change-detection task. Experiment 1 manipulated sampling cost to increase reliance on internal memory storage, whereas Experiment 2 manipulated time pressure to increase demands for maintaining consistent memory use. Increasing sampling cost led participants to use more memory but did not make individual differences in WM capacity more apparent in mean memory usage. In contrast, time pressure revealed a reliable association between WM capacity and the consistency of memory usage across sampling episodes. Higher-capacity individuals exhibited more consistent memory use. These findings suggest that WM capacity shapes voluntary memory use not simply by increasing how much information people remember, but by influencing how consistently they regulate memory use under different task demands.

## 1. Introduction

### 1.1. Working Memory Capacity and Natural Behavior

Working memory (WM) capacity is one of the core predictors of individual differences in higher cognitive abilities, including fluid intelligence, reasoning, reading comprehension, learning, and complex decision making (Conway et al., 2003; Draheim et al., 2024; Engle, 2002; Kane & Engle, 2002; Sana & Fenesi, 2025; Unsworth et al., 2021; Zhou et al., 2026). Consequently, understanding how WM capacity contributes to behavior has become an important question in cognitive science.

Traditionally, WM capacity has been conceptualized as a structural limit on the amount of information that can be simultaneously maintained (Baddeley, 1992, 2012; Cowan, 2001; Luck & Vogel, 1997; Miller, 1956). More recently, accumulating evidence suggests that WM capacity is also closely related to attentional control, reflecting the ability to maintain goal-relevant representations, resist distraction, and support goal-directed cognition (Draheim et al., 2024; Duan et al., 2026; Engle, 2002; Unsworth et al., 2021). Together, these perspectives indicate that WM capacity contributes not only to information maintenance but also to adaptive cognitive behavior.

Despite extensive evidence linking WM capacity to higher cognition, relatively little is known about how these individual differences influence behavior in natural tasks. Most evidence comes from laboratory paradigms in which memory demands are externally specified and participants are instructed to remember as much information as possible. By contrast, most everyday activities allow individuals to determine how much information to encode and maintain according to ongoing task demands. How individuals with different WM capacities regulate memory use under such conditions remains largely unknown.

### 1.2. Voluntary Memory Usage in Natural Tasks

In many natural contexts, individuals are not required to maximize memory usage. Instead, they can flexibly trade internal memory for repeated sampling from the external environment (Hayhoe, 2000; Van der Stigchel, 2020). When copying a visual display, for example, one may repeatedly look back at the model rather than encode all elements at once. In such settings, memory usage reflects voluntary decisions about how much information to encode during each sampling episode.

The copying task provides a controlled paradigm for investigating this voluntary regulation (Ballard et al., 1995). Participants reproduce a stimulus configuration from a model display in a separate workspace and may freely alternate between viewing the model and placing items. Memory usage is defined as the number of items encoded and reproduced following a single sampling of the model display. This measure captures memory usage during each sampling episode rather than the total amount of information encoded across an entire trial.

Prior work using this paradigm has consistently shown that mean memory usage per sampling episode is substantially lower than typical estimates of WM capacity (e.g., Ballard et al., 1995; Draschkow et al., 2021; Kvitelashvili & Kessler, 2024). Moreover, memory usage systematically increases as external sampling becomes more costly (for a review, see Qing et al., 2025). Similar adaptive patterns have also been observed in young children (Blaser & Kaldy, 2025; Liang et al., 2025), suggesting that flexible regulation of memory usage emerges early in development. Together, these findings indicate that natural memory behavior reflects adaptive regulation rather than simply maximizing memory storage.

### 1.3. How WM Capacity Shapes Voluntary Memory Use

Although memory usage is voluntarily regulated, WM capacity may still influence behavior in different ways.

From the perspective of memory storage, individuals with higher WM capacity should be able to encode and maintain more information following each sampling episode. In the copying task, each visit to the model creates an opportunity to encode multiple items before returning to the workspace. Higher-capacity individuals should therefore reproduce more items following each sampling, resulting in greater mean memory usage.

However, voluntary memory usage can also be viewed as an adaptive decision process. During each sampling episode, individuals determine how much information to encode by balancing the costs of maintaining information internally against the costs of acquiring information from the environment. Successful regulation therefore depends not only on storing information, but also on maintaining task-relevant information—including current goals and the relative costs and benefits of alternative actions—to guide memory-use decisions. The attentional-control account of WM proposes that individuals with higher WM capacity are better able to maintain such goal-relevant representations over time, thereby supporting more stable goal-directed behavior (Draheim et al., 2024; Engle, 2002; Unsworth et al., 2021). Consistent with this view, higher WM capacity has been associated with lower exploration under uncertainty (Laureiro-Martinez et al., 2019) and more efficient information sampling (Wan & Sloutsky, 2026). If similar mechanisms operate during voluntary memory usage, higher-capacity individuals should be less likely to make unnecessary adjustments when task demands remain stable, resulting in greater consistency in memory usage across sampling episodes.

These perspectives therefore make different predictions regarding how WM capacity shapes voluntary memory use. Individual differences may be reflected either in the amount of memory used during each sampling episode, or in the consistency with which memory is used across sampling episodes.

### 1.4. Task Structure and Voluntary Memory Use

How WM capacity shapes voluntary memory use may depend on task structure. In natural tasks, the flexibility to regulate memory use may obscure individual differences arising from both storage capacity and attentional control.

From a storage perspective, natural tasks allow individuals to trade internal memory for external sampling. Rather than approaching their storage limits, individuals can compensate for limited memory by sampling the environment more frequently. Consequently, differences in storage capacity may not translate into observable differences in memory usage. Consistent with this possibility, previous work using the copying task found little association between WM capacity and mean memory usage (Kvitelashvili & Kessler, 2024). Increasing sampling cost provides a way to reduce this compensation. As repeated sampling becomes more costly, relying on internal memory becomes increasingly advantageous, encouraging participants to use more memory and making storage-related individual differences more likely to emerge.

Behavioral flexibility may similarly obscure individual differences related to attentional control. Just as natural tasks do not require individuals to fully utilize their storage capacity, they may also place relatively weak demands on the sustained maintenance of goal-relevant information. When individuals have sufficient time, they can flexibly adjust memory usage across sampling episodes without maintaining a stable pattern of behavior. Under such conditions, differences associated with attentional control may therefore be less apparent. Time pressure provides a complementary manipulation by limiting opportunities for behavioral adjustment (e.g., Bobadilla-Suarez & Love, 2018; Bröder, 2003; Payne et al., 1988). As opportunities for adjustment decrease, maintaining a stable pattern of memory usage becomes increasingly important. If higher WM capacity supports the sustained maintenance of goal-relevant information, higher-capacity individuals should be better able to maintain consistent memory usage under time pressure, making differences in memory-use consistency more apparent.

### 1.5. The Present Study

Across two experiments, participants performed a copying task that allowed voluntary regulation of memory usage. In Experiment 1 (N = 60), we manipulated sampling cost to examine whether increasing reliance on internal memory reveals associations between WM capacity and mean memory usage. In Experiment 2 (N = 60), we introduced time pressure to examine whether limiting opportunities for behavioral adjustment reveals associations between WM capacity and the consistency of memory usage.

By systematically manipulating task conditions, the present study aimed to clarify how WM capacity shapes voluntary memory use under different task conditions.

## 2. Experiment 1: Sampling Cost Manipulation Without Time Pressure

Experiment 1 examined whether increasing sampling cost would reveal individual differences in WM capacity in a natural WM task. Sampling cost was manipulated at two levels (low vs. high), while no time pressure was imposed.

### 2.1. Methods

#### 2.1.1. Participants and Design

We set the sample size as 60 to detect a medium effect size (*ρ* = 0.35) for the correlation between memory usage and WM capacity with a statistical power of 0.8, using G*Power software, version 3.1.9.7 (Faul et al., 2007). A total of 63 participants were recruited through Zhejiang University. All participants had normal or corrected-to-normal vision. All participants received monetary compensation for their participation. Participants whose estimated WM capacity or memory usage amount was more than 2.5 standard deviations from the sample mean were excluded from the analyses. Specifically, one participant was excluded because of an extremely low WM capacity estimate (K = 0.6), and two participants were excluded because their memory usage amount was extremely high (usage = 6), resulting in a final sample of 60 participants (31 females, age range = 18–29 years). The exclusion criteria were not preregistered. All primary analyses were repeated using the full sample of 63 participants as sensitivity tests.

#### 2.1.2. Apparatus and Stimuli

Stimuli were presented on a computer screen (24-inch, 1920 × 1080, 60 Hz) with a gray background grid (7 × 21 cells). The display consisted of a gray grid (7 × 21 cells) containing three black-framed regions arranged from left to right: a model region, a workspace region, and a resource region. Each region consisted of 3 × 3 cells. All bars were orange (RGB: 255, 170, 0) and identical in size. Bar orientations were selected from nine evenly spaced values between 0° and 180°. Six bars were presented in the model region. Their locations were randomly selected without replacement from the available grid cells within the region. The resource region contained nine bars, six of which matched the orientations of the model bars and three of which were distractors. Distractor orientations were drawn from the same orientation set but were not used in the model array on that trial. The spatial arrangement of bars in the resource region was randomized and independent from that in the model region. Orientations and locations were randomly assigned for each trial. The workspace region was initially empty.

Participants interacted with the task using a standard keyboard. Cursor movement was controlled via the arrow keys. When the cursor was located in the workspace region, bars in the resource region could be copied into the workspace using nine letter keys mapped spatially to the resource bars. From left to right and top to bottom, the bars corresponded to the keys q, w, e, a, s, d, z, x, c. Pressing a key generated a copy of the corresponding bar in the grid cell occupied by the cursor.

#### 2.1.3. Experimental Task and Procedure

(1) Copying Task

**Figure 1:**
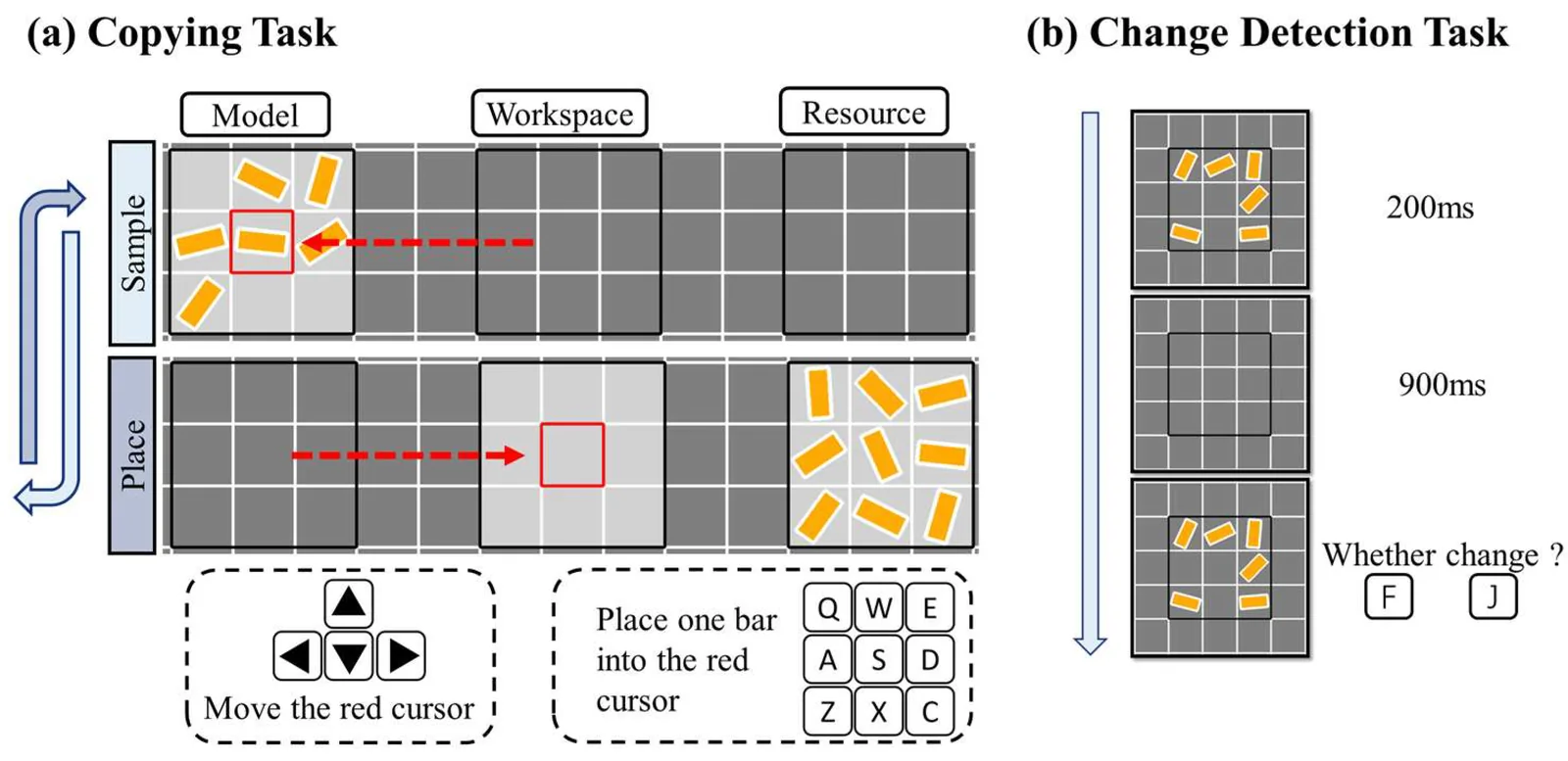
Schematic illustration of the copying task and change detection task. (**a**) Copying task. Participants reproduced a configuration of bars from a model region into a separate workspace region. Model information was visible only when the cursor was positioned within the model region; when the cursor moved to the workspace region, the model was occluded and participants had to rely on working memory to place bars selected from a resource region. Memory usage was defined as the number of bars placed following a single visit to the model region. Sampling cost was manipulated by varying the spatial distance between the model and workspace regions. (**b**) Change detection task. Each trial began with a fixation cross, followed by a memory array of six oriented bars. After a brief retention interval, a test array was presented that was either identical to the memory array or differed in the orientation of one bar. Participants indicated whether a change occurred.

Participants performed a copying task requiring selective sampling and voluntary use of working memory. An example of the display is shown in Figure 1a.

Participants used the arrow keys to move a red cursor across the display. Model visibility depended on cursor location: when the cursor was in the model region, only the model bars were visible; when the cursor was in the workspace region, bars in the workspace and resource regions were visible, while the model bars were hidden. This design required participants to actively sample information from the model region and rely on WM during reconstruction. While in the workspace region, participants could copy a bar from the resource region into the current grid cell by pressing a corresponding letter key. Participants could place any number of bars before returning to the model region.

Once a bar was placed, its position was occluded by a black feedback panel whenever the cursor entered the model region. Correct placements were indicated by a score of 5 points, whereas incorrect placements were marked with an “X.” Trials ended when participants placed six bars in the workspace and pressed the space bar. After a 1 s delay, the display was cleared and the next trial began.

Sampling cost was manipulated by varying the distance between the centers of the model and workspace regions. In the low-cost condition, the distance was five grid cells; while in the high-cost condition it was eight grid cells. Sampling cost was manipulated using a blocked design, with 20 trials per block, and block order was counterbalanced across participants.

(2) Change Detection Task

Working memory capacity was assessed using a change detection task, with stimuli comparable to those in the copying task (see Figure 1b). Bars were presented within a 3 × 3 grid centered on the screen. In each trial, six grid locations were randomly selected without replacement to contain bars. Orientations were selected from nine evenly spaced values between 0° and 180° and were randomly assigned to the six memory bars in each trial.

The task procedure was identical to that reported in Balaban et al. (2019). The memory array, consisting of six bars, was presented for 200 ms and then removed. After a 900 ms retention interval, the test array was displayed. The test array was either identical to the memory array or differed in the orientation of one bar. In change trials, one bar was replaced by a bar whose orientation was sampled from the remaining values in the orientation set, resulting in orientation changes of ±20°, ±40°, ±60°, or ±80° relative to the original orientation.

Participants indicated whether the test array was the same as or different from the memory array by pressing the “F” or “J” key. The task consisted of 60 trials. Change and no-change trials occurred with equal probability. The location of the changed bar was randomized across trials.

### 2.2. Data Analysis

All analyses were conducted in Python (Version 3.12.4; Van Rossum & Drake, 2009), using the packages pingouin (Version 0.5.4; Vallat, 2018) and seaborn (Version 0.13.2; Waskom, 2021). Analyses focused on whether individual differences in WM capacity were associated with systematic differences in memory allocation behavior in the copying task.

#### 2.2.1. Estimation of WM Capacity

WM capacity was estimated from performance in the change detection task using Cowan’s (2001) formula: K = N × (HIT − FA), where N denotes the set size, HIT represents the hit rate (proportion of correct responses on change trials), and FA represents the false-alarm rate (proportion of incorrect responses on no-change trials). This estimate served as the primary index of individual differences in WM capacity.

#### 2.2.2. Quantification of Memory Usage in the Copying Task

Memory usage was quantified at the level of individual sampling episodes. For each sampling episode, memory usage was defined as the number of bars encoded and reproduced following a single sampling of the model display. Episodes in which no bars were placed were excluded, as they did not yield measurable memory output and were likely to reflect accidental actions or aborted sampling attempts.

To capture distinct aspects of memory usage, two types of indices were derived.

(1) Memory Usage Amount

According to our definition of memory usage, the amount of memory used during each sampling episode can be inferred from the number of items participants attempted to reproduce after a single visit to the model display. Accordingly, memory usage amount was quantified as the average number of items reproduced following each sampling episode. Because this measure includes both successful retrieval and placement attempts based on incomplete memory, we additionally calculated correct memory usage, defined as the average number of correctly reproduced items following each sampling episode. This measure provides a more conservative estimate of the amount of information successfully maintained and used from working memory.

(2) Memory Usage Entropy

To characterize the consistency of memory use within individuals, Shannon entropy was computed for each participant’s distribution of memory usage across sampling episodes. Entropy captures the overall dispersion of allocation choices, with lower values indicating more concentrated and stable usage patterns, and higher values reflecting more variable usage across usage levels. Entropy was used instead of standard deviation (SD) because memory usage is discrete and often non-normally distributed; entropy quantifies distributional concentration without assuming symmetry or linear distance between usage levels (Gell-Mann & Lloyd, 1996; Shannon, 1948).

To examine whether the effects observed with entropy were robust, we additionally calculated the conventional SD of memory usage across sampling episodes for each participant. Higher SD indicates greater variability in memory usage. The relationship between WM capacity and this variability measure was examined using the same analytical approach as the entropy analysis.

#### 2.2.3. Statistical Analysis

Associations between WM capacity and memory-usage measures were assessed using Spearman rank-order correlations to guarantee robustness to non-normality. All correlation tests were two-tailed. Multiple comparisons were corrected using the Holm method (Holm, 1979).

To examine whether the relationship between WM capacity and memory usage differed across sampling cost conditions, correlation coefficients obtained under low and high sampling cost were compared using a non-parametric bootstrap procedure (Efron & Tibshirani, 1994). The bootstrap procedure resampled the participant data with replacement for 5000 times to derive empirical *p*-values using the centered bootstrap method (Davison & Hinkley, 1997).

As a conventional manipulation check, we tested whether increasing sampling cost led to higher mean memory usage per sampling episode. We applied a repeated-measures ANOVA and reported the partial eta square ($\eta_{p}^{2}$) as effect size.

### 2.3. Results

#### 2.3.1. Manipulation Check for Sampling Cost

**Figure 2:**
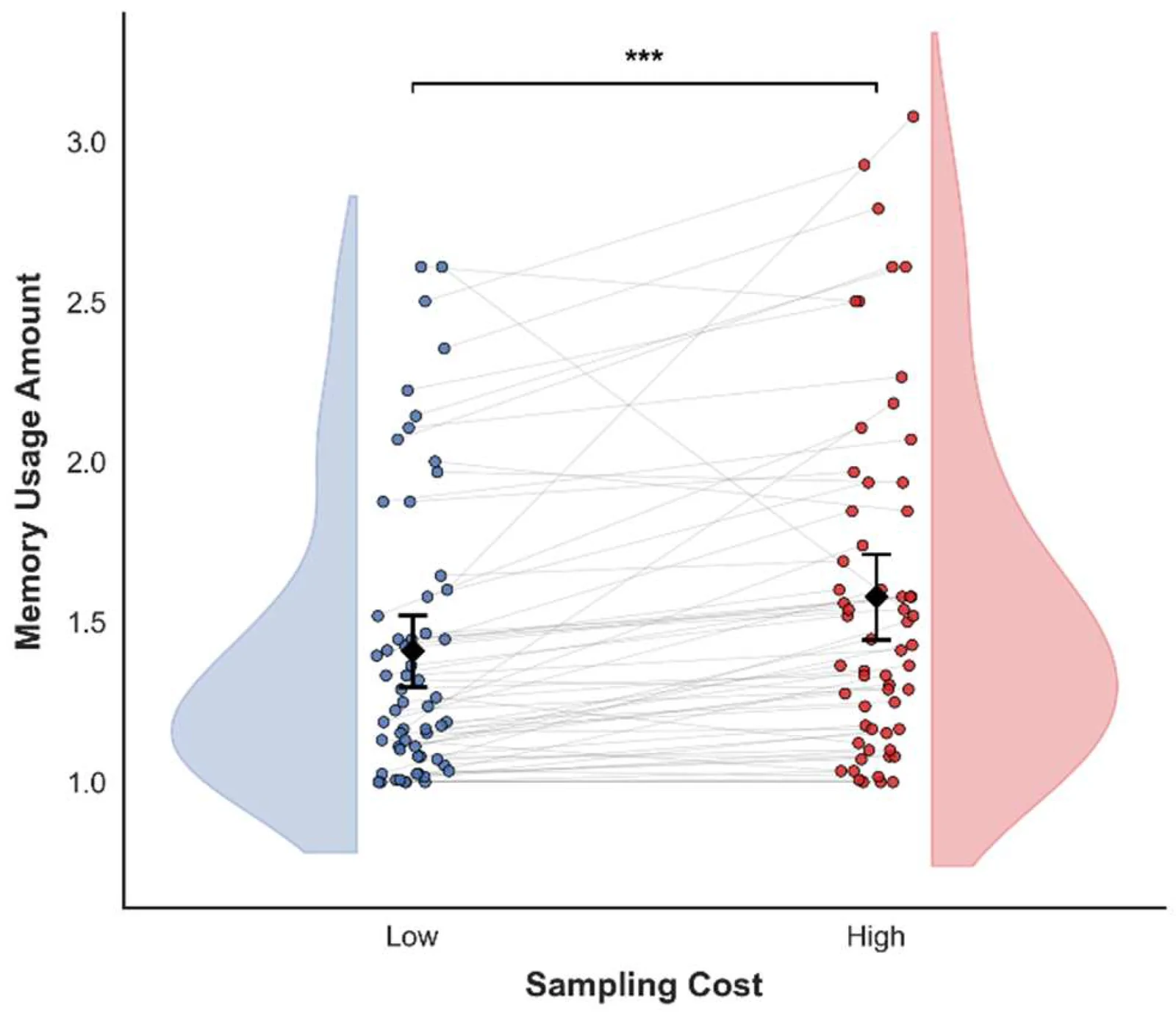
Effect of sampling cost on memory usage amount from Experiment 1. Mean memory usage per sampling episode in the low- and high-cost conditions. The small connected (blue and red) dots represent individual participants’ means, the large diamonds represent group means, and the error bars represent 95% confidence intervals. The split violin plots show the spread of the participants’ means. Significance levels: *** *p* < 0.001.

A repeated-measures ANOVA showed a significant effect of sampling cost on memory usage amount (see Figure 2), *F*(1, 59) = 17.390, *p* < 0.001, $\eta_{p}^{2}$ = 0.228. Memory usage amount was lower in the low-cost condition (M = 1.409, SD = 0.444) than in the high-cost condition (M = 1.578, SD = 0.529). Analyses based on the correct memory usage yielded the same pattern of results, *F*(1, 59) = 14.482, *p* < 0.001, $\eta_{p}^{2}$ = 0.197, confirming the robustness of the findings.

#### 2.3.2. Working Memory Capacity and Memory Usage Amount

**Figure 3:**
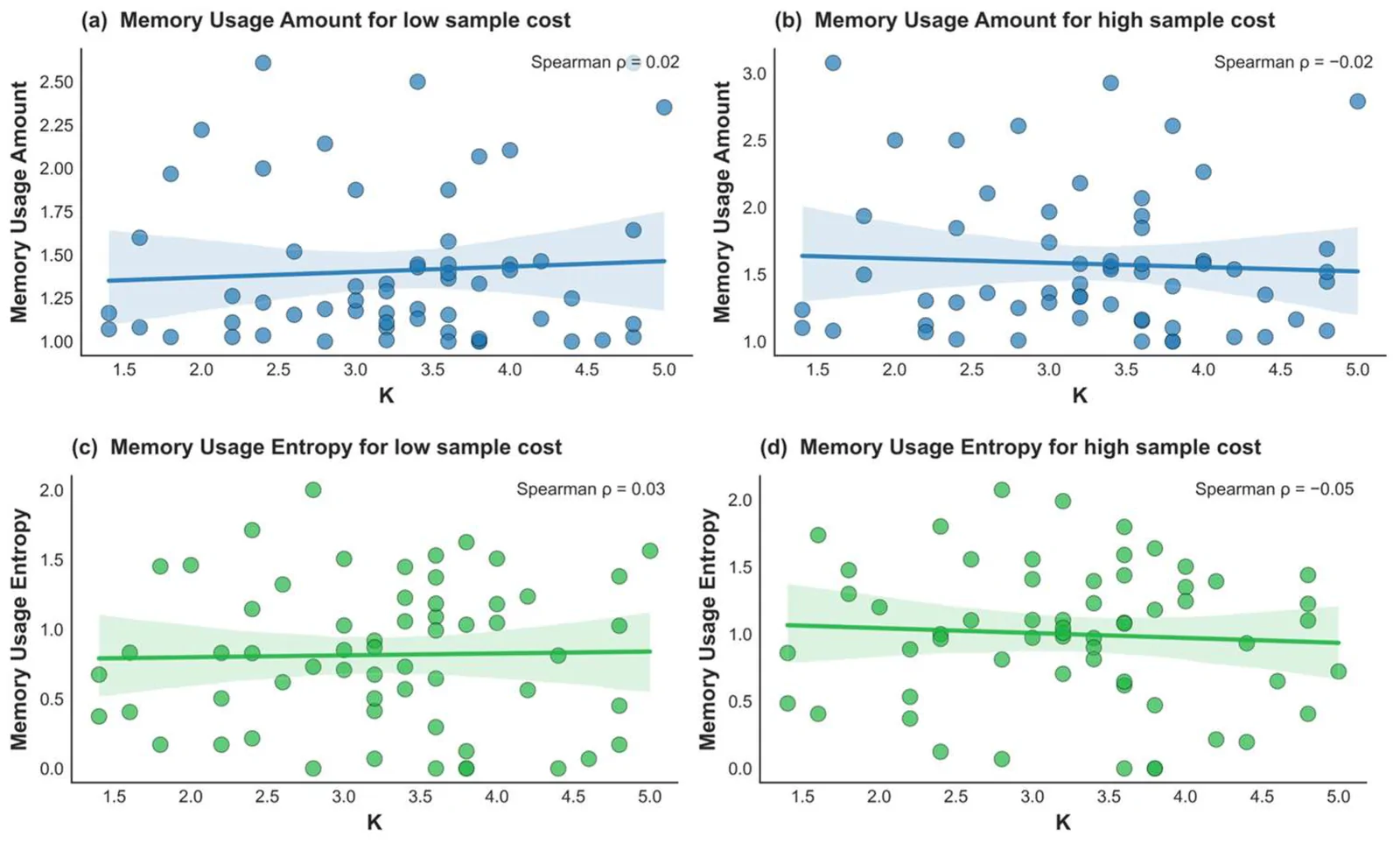
Individual differences in WM capacity (K) and memory usage from Experiment 1. Panel (**a**) shows the relationship between WM capacity (K) and memory usage amount under low sampling cost. Panel (**b**) shows the relationship between WM capacity (K) and memory usage amount under high sampling cost. Panel (**c**) shows the relationship between WM capacity (K) and memory usage entropy under low sampling cost. Panel (**d**) shows the relationship between WM capacity (K) and memory usage entropy under high sampling cost. Each point represents one participant. Solid lines represent least-squares regression fits, and shaded areas surrounding the regression lines represent 95% confidence intervals.

K was not significantly associated with memory usage amount in either the low-cost condition, *Spearman ρ* = 0.018, *p_(Holm)_* = 1.000, 95% CI [−0.237, 0.271], or the high-cost condition, *Spearman ρ* = −0.019, *p_(Holm)_* = 0.885, 95% CI [−0.272, 0.236] (see Figure 3). The same analyses based on the correct memory usage showed the same pattern (all *ps* > 0.959). Bootstrap procedure (5000 iterations) showed that the correlation coefficients did not differ significantly between low and high sampling cost conditions (for coefficients between K and memory usage amount, Δ*ρ* = 0.037, *p_(Holm)_* = 0.610; for coefficients between K and correct memory usage, Δ*ρ* = 0.067, *p_(Holm)_* = 0.804).

#### 2.3.3. Working Memory Capacity and Memory Usage Entropy

For memory usage entropy, no significant associations were observed in either the low-cost condition, *Spearman ρ* = 0.029, *p_(Holm)_* = 0.827, 95% CI [−0.227, 0.281], nor the high-cost condition, *Spearman ρ* = −0.054, *p_(Holm)_* = 1.000, 95% CI [−0.303, 0.203]. Consistent with the entropy analysis, K was not significantly associated with the SD of memory usage (all *ps* > 0.969). Bootstrap procedure (5000 iterations) showed that the correlation coefficients did not differ significantly between low and high sampling cost conditions (for coefficients between K and memory usage entropy, Δ*ρ* = 0.082, *p_(Holm)_* = 0.861; for coefficients between K and memory usage SD, Δ*ρ* = 0.005, *p_(Holm)_* = 0.950).

#### 2.3.4. Sensitivity Tests

Sensitivity test including all 63 participants yielded the same overall pattern of results as the primary analyses. K was not significantly associated with memory usage amount (all *ps* > 0.801), nor the overall memory usage entropy (all *ps* > 0.930). These results indicated that the main findings were not dependent on the exclusion of participants with extreme WM capacity or memory usage amount.

Together, the results of Experiment 1 indicate that manipulating sampling cost alone did not reveal individual differences in working memory capacity in either the amount or the consistency of memory usage.

## 3. Experiment 2: Sampling Cost Manipulation Under Time Pressure

Experiment 2 examined whether introducing explicit time pressure would promote the relationship between WM capacity and natural memory use. As in Experiment 1, sampling cost was manipulated at two levels, but each trial was subject to a fixed time limit.

### 3.1. Methods

#### 3.1.1. Participants and Design

Another 63 participants were recruited through Zhejiang University. All participants had normal or corrected-to-normal vision. All participants received monetary compensation for their participation. Participants whose estimated WM capacity or memory usage amount was more than 2.5 standard deviations from the sample mean were excluded from the analyses. Specifically, two participants were excluded because their WM capacity estimates were extremely low (both K = 0.4), and one participant was excluded because of an extremely high memory usage amount (usage = 6), resulting in a final sample of 60 participants (36 females, age range = 17–30 years). All primary analyses were repeated using the full sample of 63 participants as sensitivity analyses.

#### 3.1.2. Stimuli and Procedure

The stimuli and procedure in Experiment 2 were the same as in Experiment 1, except that each trial was forced to terminate after 30 s if the whole model was not completed in the copying task. Based on participants’ average trial completion time in Experiment 1 (around 40 s), a 30 s time limit was expected to effectively constrain behavioral adjustments.

### 3.2. Data Analysis

The data analysis in Experiment 2 was consistent with that in Experiment 1.

### 3.3. Results

#### 3.3.1. Manipulation Check for Sampling Cost

**Figure 4:**
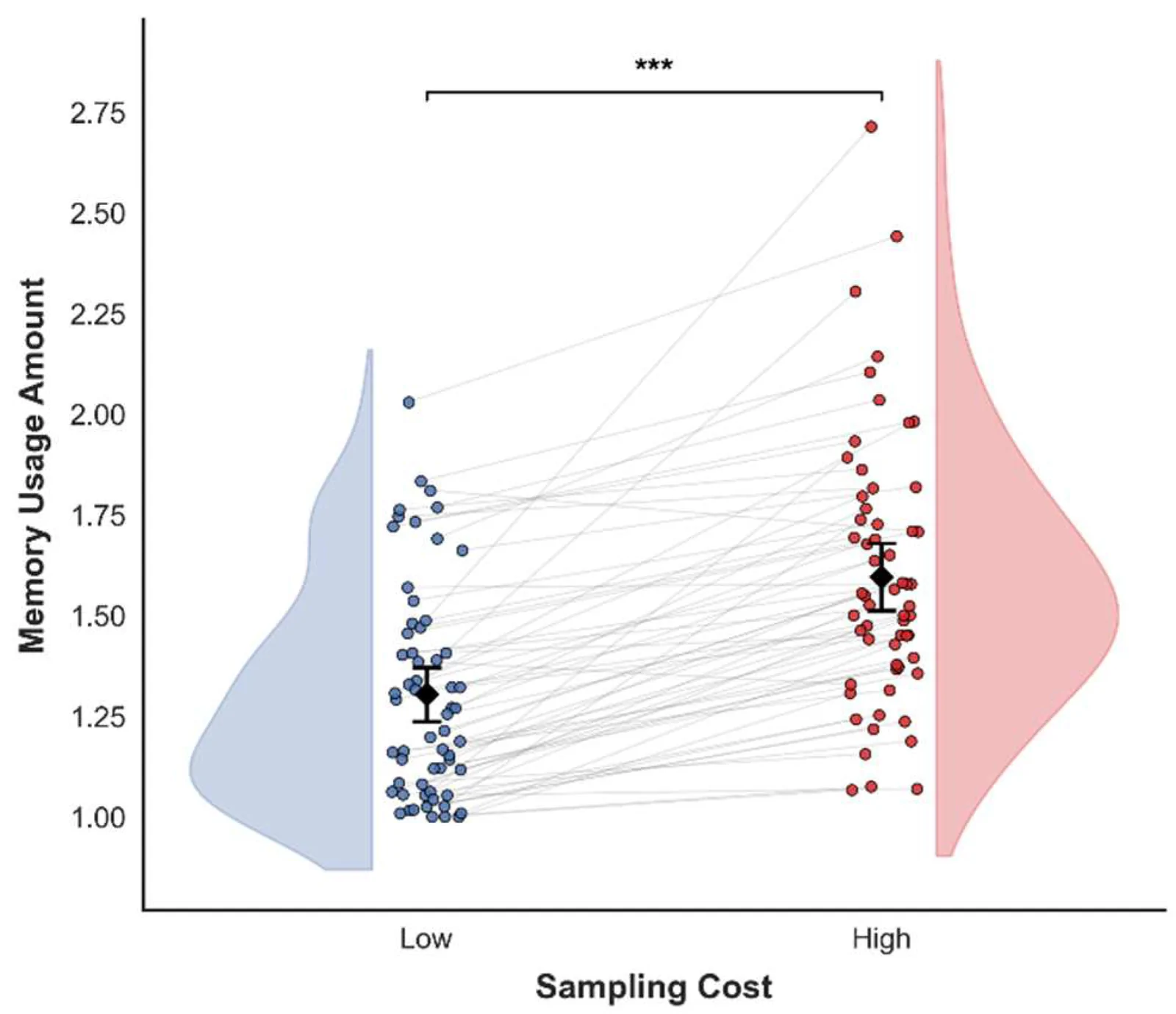
Effect of sampling cost on memory usage amount from Experiment 2. Mean memory usage per sampling episode in the low- and high-cost conditions. The small connected (blue and red) dots represent individual participants’ means, the large diamonds represent group means, and the error bars represent 95% confidence intervals. The split violin plots show the spread of the participants’ means. Significance levels: *** *p* < 0.001.

A repeated-measures ANOVA showed a significant effect of sampling cost on memory usage amount (see Figure 4), *F*(1, 59) = 97.243, *p* < 0.001, $\eta_{p}^{2}$ = 0.622. Memory usage amount was lower in the low-cost condition (M = 1.304, SD = 0.264) than in the high-cost condition (M = 1.596, SD = 0.330). Analyses based on the correct memory usage yielded the same pattern of results, *F*(1, 59) = 89.293, *p* < 0.001, $\eta_{p}^{2}$ = 0.602.

#### 3.3.2. Working Memory Capacity and Memory Usage Amount

**Figure 5:**
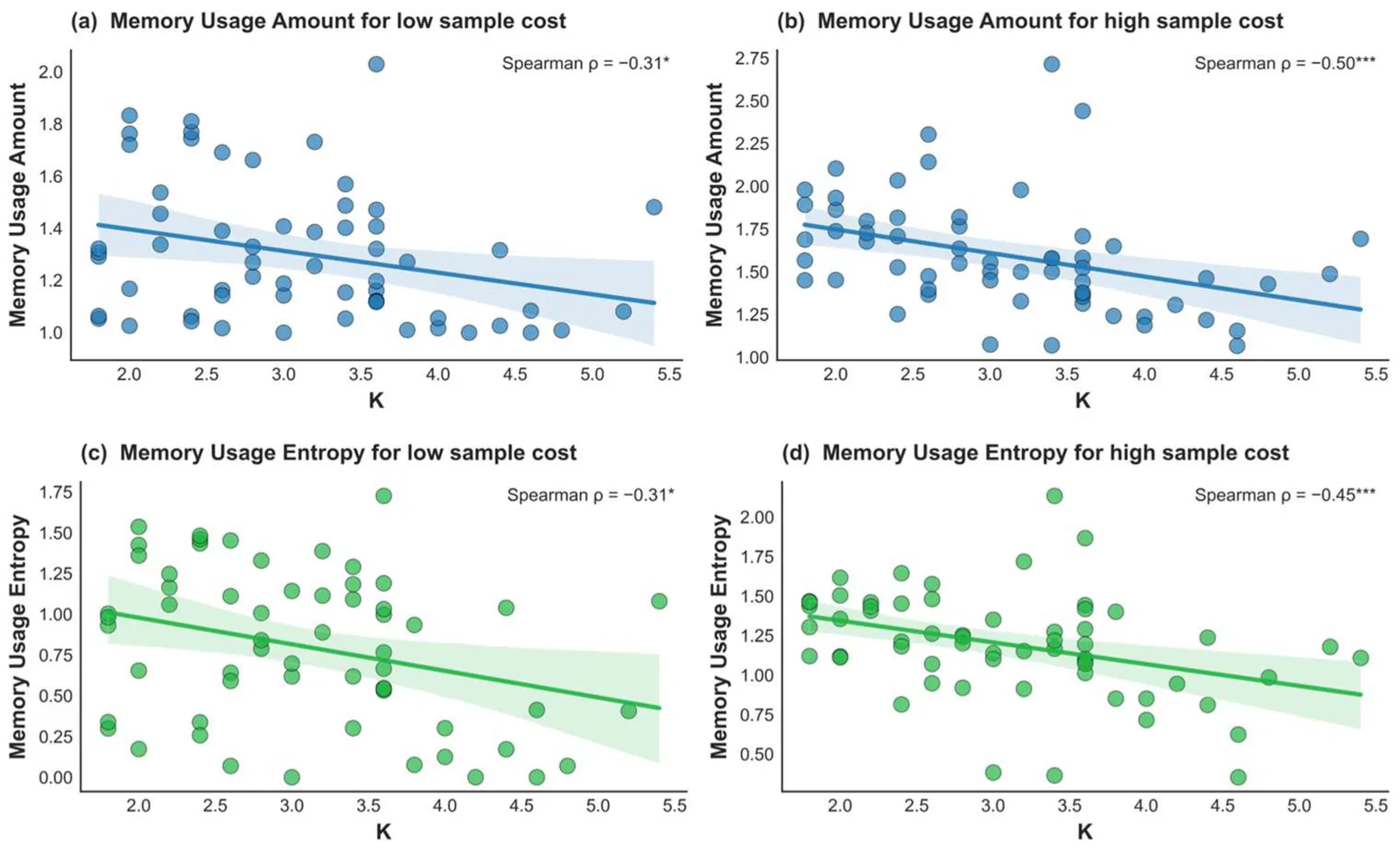
Individual differences in WM capacity (K) and memory usage from Experiment 2. Panel (**a**) shows the relationship between WM capacity (K) and memory usage amount under low sampling cost. Panel (**b**) shows the relationship between WM capacity (K) and memory usage amount under high sampling cost. Panel (**c**) shows the relationship between WM capacity (K) and memory usage entropy under low sampling cost. Panel (**d**) shows the relationship between WM capacity (K) and memory usage entropy under high sampling cost. Each point represents one participant. Solid lines represent least-squares regression fits, and shaded areas surrounding the regression lines represent 95% confidence intervals. Significance levels: * *p* < 0.05, *** *p* < 0.001.

K was significantly associated with memory usage amount in both sampling cost conditions (see Figure 5; for low-cost condition, *Spearman ρ* = −0.311, *p_(Holm)_* = 0.016, 95% CI [−0.523, −0.061]; for high-cost condition, *Spearman ρ* = −0.503, *p_(Holm)_* < 0.001, 95% CI [−0.671, −0.285]). The corresponding analyses on the correct memory usage showed the same pattern in both sampling cost conditions (for the low-cost condition, *Spearman ρ* = −0.258, *p_(Holm)_* = 0.047, 95% CI [−0.480, −0.004]; for the high-cost condition, *Spearman ρ* = −0.346, *p_(Holm)_* = 0.014, 95% CI [−0.552, −0.101]). Bootstrap procedure (5000 iterations) showed that the correlation coefficients did not differ significantly between low and high sampling cost conditions (for coefficients between K and memory usage amount, Δ*ρ* = 0.192, *p_(Holm)_* = 0.091; for coefficients between K and correct memory usage, Δ*ρ* = 0.089, *p_(Holm)_* = 0.376).

#### 3.3.3. Working Memory Capacity and Memory Usage Entropy

K was significantly related to the memory usage entropy in both sampling cost conditions (see Figure 5; for low-cost condition, *Spearman ρ* = −0.310, *p_(Holm)_* = 0.016, 95% CI [−0.523, −0.061]; for high-cost condition, *Spearman ρ* = −0.452, *p_(Holm)_* < 0.001, 95% CI [−0.633, −0.223]). Consistent with the entropy analysis, K was significantly associated with the SD of memory usage (for low-cost condition, *Spearman ρ* = −0.280, *p_(Holm)_* = 0.030, 95% CI [−0.498, −0.028]; for high-cost condition, *Spearman ρ* = −0.341, *p_(Holm)_* = 0.015, 95% CI [−0.547, −0.095]), indicating that the relationship between WM capacity and behavioral consistency was not specific to entropy as a measure of behavioral consistency. Bootstrap procedure (5000 iterations) showed that the correlation coefficients did not differ significantly between low and high sampling cost conditions (for coefficients between K and memory usage entropy, Δ*ρ* = 0.142, *p_(Holm)_* = 0.448; for coefficients between K and memory usage SD, Δ*ρ* = 0.061, *p_(Holm)_* = 0.616).

#### 3.3.4. Sensitivity Tests

Sensitivity test including all 63 participants yielded the same overall pattern of results as the primary analyses. K was significantly associated with memory usage amount (for low-cost condition, *Spearman ρ* = −0.323, *p* = 0.010, 95% CI [−0.528, −0.082]; for high-cost condition, *Spearman ρ* = −0.468, *p* < 0.001, 95% CI [−0.641, −0.249]). K was also significantly associated with memory usage entropy (for the low-cost condition, *Spearman ρ* = −0.385, *p* = 0.002, 95% CI [−0.578, −0.152]; for the high-cost condition, *Spearman ρ* = −0.454, *p* < 0.001, 95% CI [−0.631, −0.233]). These results indicated that the main findings were not dependent on the exclusion of participants with extreme WM capacity or memory usage amount.

#### 3.3.5. Additional Analysis: Concentrated Memory Usage

Because entropy indicates the overall concentration of the memory usage distribution but does not identify which usage level contributes to this concentration, we conducted an additional analysis to characterize the dominant memory usage pattern.

**Figure 6:**
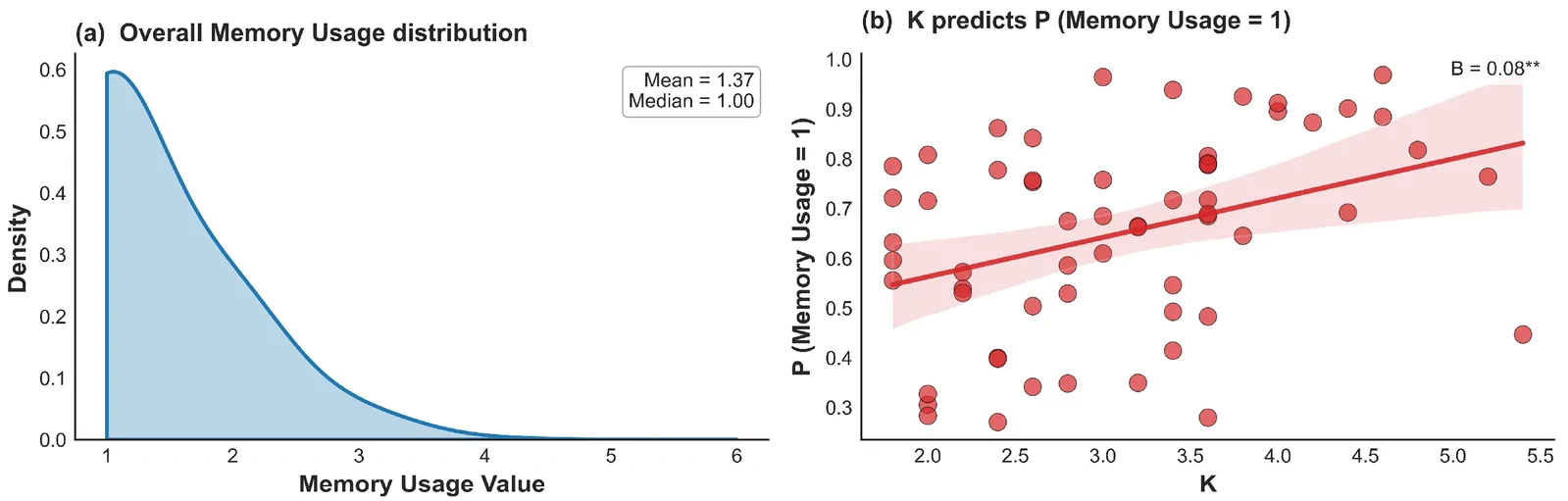
The concentrated memory usage from Experiment 2. Panel (**a**) shows the Kernel density estimate of memory usage across all trials and participants. The distribution is right-skewed, with a mean of 1.37 and a median of 1. Panel (**b**) shows the relationship between individual working memory capacity (K) and the proportion of episodes in which the memory usage value was exactly one. Each point represents one participant. The red line indicates the best-fit regression line with 95% confidence interval (shaded band). Significance levels: ** *p* < 0.01.

To characterize the overall distribution of memory usage, we plotted kernel density estimates across all participants (see Figure 6a). The distribution was strongly concentrated at a memory usage of one item, indicating that using one item per sampling episode was the dominant behavioral pattern under time pressure.

To further examine whether WM capacity was associated with the tendency to adopt the dominant memory-use pattern, we conducted a regression analysis treating WM capacity as a continuous predictor. For each participant, we calculated the proportion of sampling episodes in which memory usage was exactly one item. A linear regression revealed that WM capacity significantly predicted the proportion, *B* = 0.079, 95% CI [0.026, 0.132], *t*(58) = 2.93, *p* = 0.004, *R*^2^ = 0.132 (see Figure 6b).

Thus, the greater concentration of memory usage among higher-capacity individuals was accompanied by a greater tendency to adopt the dominant one-item memory usage pattern.

## 4. Discussion

### 4.1. Overview of the Findings

The present study investigated how WM capacity shapes voluntary memory use in natural tasks in which individuals freely determine how much information to encode into internal storage. Across two Experiments, we found that the influence of WM capacity depended critically on task structure. Increasing sampling cost encouraged participants to encode more information following each sampling episode but did not make individual differences in WM capacity more apparent in mean memory usage. In contrast, imposing time pressure revealed reliable associations between WM capacity and the consistency of memory usage across sampling episodes, while higher-capacity individuals also exhibited lower mean memory usage.

Together, these findings suggest that WM capacity does not simply determine how much information people maintain during natural behavior. Instead, its influence depends on the constraints imposed by the task.

### 4.2. Task Structure Influences How WM Capacity Shapes Voluntary Memory Use

A central finding of the present study is that task structure influenced not only memory use itself but also how WM capacity was reflected in behavior.

Increasing sampling cost produced the expected increase in memory usage per sampling episode, replicating previous findings from the copying paradigm (e.g., Ballard et al., 1995; Draschkow et al., 2021; Qing et al., 2025). When repeated sampling became more costly, participants encoded more information before returning to the workspace, demonstrating adaptive regulation of memory use in response to environmental demands. However, increasing reliance on internal memory did not strengthen the relationship between WM capacity and mean memory usage. Even when external sampling became more expensive, participants retained considerable flexibility in determining how much information to encode following each sampling episode. Individuals with higher WM capacity could therefore compensate by sampling the model more frequently, allowing similar observable memory usage despite differences in underlying capacity.

Time pressure produced a qualitatively different pattern. Unlike sampling cost, which alters the relative costs and benefits of internal memory and external sampling, time pressure limits opportunities to adjust behavior during task performance. Under these conditions, higher-capacity individuals exhibited significantly greater consistency in memory usage across sampling episodes.

Time pressure may increase the influence of general task-efficiency factors, such as processing speed or motor efficiency. Although these factors may facilitate faster execution, they do not readily explain why higher-capacity individuals showed greater consistency in memory regulation across sampling episodes. Time pressure may also encourage more conservative memory-use strategies, as reducing the amount of information encoded during each sampling episode may reduce the risk that an ongoing memory operation be interrupted before the deadline. However, such an account does not explain the direction of the present pattern. If conservative memory use primarily reflected avoiding capacity-related failures, it would be expected to be more relevant for lower-capacity individuals, whose ability to maintain information is more limited.

A more theoretically relevant interpretation is that higher-capacity individuals might be better able to maintain a stable memory-use policy throughout each trial. Rather than repeatedly adjusting how much information to encode after successive visits to the model display, they may have more consistently followed an effective memory-use policy once task demands were established. Although the present study did not directly measure policy selection or policy stability, this interpretation is consistent with evidence that WM capacity supports the maintenance of goal-relevant information for guiding adaptive behavior (Draheim et al., 2024; Engle, 2002; Laureiro-Martinez et al., 2019; Unsworth et al., 2021; Wan & Sloutsky, 2026). The present findings extend this perspective by suggesting that WM capacity may likewise contribute to maintaining stable memory-use policy during natural tasks.

Other mechanisms may also contribute to this behavioral consistency. For example, repeated successful regulation may eventually give rise to efficient cognitive routines that reduce the need for continuous online adjustment. This possibility is conceptually related to research on automatization, which suggests that well-established cognitive routines can reduce working memory demands and reliance on continuous control during complex performance (Fabio et al., 2026). Under demanding conditions such as time pressure, reliance on such efficient routines may become particularly important because opportunities for online adjustment are limited. Future studies will be needed to determine whether consistent memory regulation in natural tasks primarily reflects attentional-control processes, automatized routines, or their interaction.

Interestingly, higher-capacity individuals also showed lower mean memory usage under time pressure (Experiment 2). This finding was not the primary focus of Experiment 2, which was designed to examine whether WM capacity was associated with the consistency of memory use under time pressure. Moreover, this pattern is opposite to the prediction derived from a memory-storage account, which would suggest that higher-capacity individuals should use more memory. Reducing memory usage would appear inefficient from this perspective because it would diminish the potential advantage afforded by greater storage capacity. A possible explanation for this pattern comes from the distributional characteristics of memory use. Previous studies have shown that when sampling costs are moderate, encoding approximately one or two items per sampling episode is typically the dominant behavioral pattern (Ballard et al., 1995; Draschkow et al., 2021; Kvitelashvili & Kessler, 2024). Because memory usage distributions in the present study were similarly concentrated around these lower values, greater consistency naturally reduced the mean memory usage. The reduction in mean memory usage therefore appears to arise from greater behavioral consistency rather than deliberate minimization of memory load.

### 4.3. Implications for Understanding Working Memory in Natural Tasks

The present findings contribute to a growing body of work suggesting that laboratory measures of WM capacity cannot be understood solely in terms of storage limitations. Traditional laboratory paradigms estimate the maximum amount of information that individuals can maintain under externally specified task demands. In contrast, many natural tasks require individuals to determine how much information should be maintained at any given moment. Understanding WM capacity in such contexts therefore requires understanding not only what people are able to remember but also how they choose to use memory.

The present results suggest that WM capacity shapes voluntary memory use primarily through the regulation of behavior rather than simply by increasing memory quantity. Participants with higher WM capacity did not consistently use more memory than lower-capacity individuals. Instead, they exhibited more consistent patterns of memory use when task conditions reduced opportunities for behavioral adjustment. This finding complements recent evidence that WM capacity predicts more efficient information sampling and reduced exploratory behavior during decision making (Laureiro-Martinez et al., 2019; Wan & Sloutsky, 2026), suggesting that WM capacity may contribute more broadly to maintaining stable goal-directed behavior across different forms of cognitive regulation.

The present findings also highlight the importance of examining distributional characteristics of behavior rather than relying solely on mean performance. Mean memory usage captures the amount of information encoded following each sampling episode, whereas the distribution of memory usage reflects how consistently individuals regulate memory across repeated decisions. This observation aligns with increasing recognition that behavioral variability contains theoretically meaningful information rather than merely reflecting measurement noise (e.g., Bays et al., 2024; Tomić et al., 2024; Tomić & Bays, 2024). Distributional analyses may therefore provide an indispensable approach for understanding how cognitive abilities influence natural behavior.

### 4.4. Limitations and Future Directions

Several limitations should be acknowledged. First, the present interpretation that higher-capacity individuals maintained more stable memory-use policies remains speculative because policy itself was not directly measured. Future work combining computational modeling, eye tracking, or process-tracing methods may help characterize the latent decision processes underlying voluntary memory use and directly test whether policy stability mediates the relationship between WM capacity and behavioral consistency.

Second, the present study only examined sampling cost and time pressure. Other task manipulations, such as uncertainty, multitasking, or changing reward structures, may reveal different aspects of how WM capacity shapes voluntary memory use during natural cognition.

## 5. Conclusions

The present study demonstrates that the relationship between WM capacity and voluntary memory use depends critically on task structure. Increasing sampling cost changed how much memory participants used but did not make individual differences in WM capacity more apparent in mean memory usage. In contrast, time pressure revealed reliable associations between WM capacity and the consistency of memory use across sampling episodes. These findings suggest that WM capacity shapes voluntary memory use not simply by determining how much information individuals maintain, but by influencing how consistently they regulate memory under task constraints. More broadly, the present study extends the investigation of WM capacity from laboratory estimates of storage limits toward understanding how memory is adaptively used during natural behavior.

## Data Availability

Experiment materials, deidentified data and data-analysis scripts for both of the experiments have been made publicly available via OSF and can be accessed at [https://osf.io/eajy7/] (accessed on 7 March 2026). Experiment 2 was preregistered.

## Abbreviations

The following abbreviations are used in this manuscript:

WM	Working memory
K	Estimated WM capacity
SD	Standard deviation
